# Genetic variation in gonadal impairment in female survivors of childhood cancer: a PanCareLIFE study protocol

**DOI:** 10.1186/s12885-018-4834-3

**Published:** 2018-09-26

**Authors:** Anne-Lotte L. F. van der Kooi, Eva Clemens, Linda Broer, Oliver Zolk, Julianne Byrne, Helen Campbell, Marleen van den Berg, Claire Berger, Gabriele Calaminus, Uta Dirksen, Jeanette Falck Winther, Sophie D Fosså, Desiree Grabow, Riccardo Haupt, Melanie Kaiser, Tomas Kepak, Leontien Kremer, Jarmila Kruseova, Dalit Modan-Moses, Andreas Ranft, Claudia Spix, Peter Kaatsch, Joop S. E. Laven, Eline van Dulmen-den Broeder, André G. Uitterlinden, Marry M. van den Heuvel-Eibrink, on behalf of the PanCareLIFE Consortium, Kylie O’Brien, Thorsten Langer, Anja Borgmann-Staudt, Antoinette am Zehnhoff-Dinnesen, Claudia Kuehni, Katja Baust, Floor van Leeuwen, Gabriele Strauss, R Haupt, M L Garré, Alison Leiper, H Lackner, Anna Panasiuk, Maryna Krawczuk-Rybak, Marina Kunstreich, Holger Cario, Stefan Bielack, Joanna Stefanowicz, Victoria Grandage

**Affiliations:** 1 Division of Reproductive Endocrinology and Infertility, Department of Obstetrics and Gynecology, Erasmus MC – Sophia Children’s Hospital, Rotterdam, The Netherlands; 2 Department of Pediatric Hematology and Oncology, Erasmus MC – Sophia Children’s Hospital, Rotterdam, The Netherlands; 3 Princess Máxima Center for Pediatric Oncology, Lundlaan 6, 3584 EA Utrecht, The Netherlands; 4 Department of Internal Medicine, Erasmus MC, Rotterdam, The Netherlands; 5 Institute of Pharmacology of Natural Products and Clinical Pharmacology, University Hospital Ulm, Ulm, Germany; 6 Boyne Research Institute, Drogheda, Ireland; 7 Department of Pediatric Hematology and Oncology, VU Medical Center, Amsterdam, The Netherlands; 8 Department of Paediatric Oncology, University Hospital, St-Etienne, France; 9 Epidemiology of Childhood and Adolescent Cancers, CRESS, INSERM, UMR 1153, Paris Descartes University, Villejuif, France; 10 Department of Paediatric Haematology and Oncology, University Children’s Hospital Bonn, University of Bonn Medical School, Bonn, Germany; 11 Pediatrics III, West German Cancer Centre, University Hospital Essen, Essen, Germany; 12 German Cancer Research Centre, DKTK, sites Bonn and Essen, Germany; 13 Danish Cancer Society Research Center, Copenhagen, Denmark; 14 Department of Clinical Medicine, Faculty of Health, Aarhus University, Aarhus, Denmark; 15 Department of Oncology, Oslo University Hospital, Oslo, Norway; 16 German Childhood Cancer Registry, Institute of Medical Biostatistics, Epidemiology and Informatics, University Medical Center, Mainz, Germany; 17 Epidemiology and Biostatistics Unit, Istituto Giannina Gaslini, Genoa, Italy; 18 Czech Republic & International Clinical Research Center (FNUSA-ICRC), University Hospital Brno, Brno, Czech Republic; 19 Department of Pediatrics, Academic Medical Center, Emma Children’s Hospital, Amsterdam, The Netherlands; 20 Motol University Hospital, Prague, Czech Republic; 21 Chaim Sheba Medical Center, The Edmond and Lily Safra Children’s Hospital, Tel Hashomer, Israel; 22 Sackler Faculty of Medicine, Tel-Aviv University, Tel-Aviv, Israel

**Keywords:** Childhood cancer survivor, Genetic variations, SNPs, Late effects, GWAS

## Abstract

**Background:**

Improved risk stratification, more effective therapy and better supportive care have resulted in survival rates after childhood cancer of around 80% in developed countries. Treatment however can be harsh, and three in every four childhood cancer survivors (CCS) develop at least one late effect, such as gonadal impairment. Gonadal impairment can cause involuntary childlessness, with serious consequences for the well-being of CCS. In addition, early menopause increases the risk of comorbidities such as cardiovascular disease and osteoporosis. Inter-individual variability in susceptibility to therapy related gonadal impairment suggests a role for genetic variation.

Currently, only one candidate gene study investigated genetic determinants in relation to gonadal impairment in female CCS; it yielded one single nucleotide polymorphism (SNP) that was previously linked with the predicted age at menopause in the general population of women, now associated with gonadal impairment in CCS. Additionally, one genome wide association study (GWAS) evaluated an association with premature menopause, but no GWAS has been performed using endocrine measurements for gonadal impairment  as the primary outcome in CCS.

**Methods:**

As part of the PanCareLIFE study, the genetic variability of chemotherapy induced gonadal impairment among CCS will be addressed. Gonadal impairment will be determined by anti-Müllerian hormone (AMH) levels or alternatively by fertility and reproductive medical history retrieved by questionnaire. Clinical and genetic data from 837 non-brain or non-bilateral gonadal irradiated long-term CCS will result in the largest clinical European cohort assembled for this late-effect study to date. A candidate gene study will examine SNPs that have already been associated with age at natural menopause and DNA maintenance in the general population. In addition, a GWAS will be performed to identify *novel* allelic variants. The results will be validated in an independent CCS cohort.

**Discussion:**

This international collaboration aims to enhance knowledge of genetic variation which may be included in risk prediction models for gonadal impairment in CCS.

## Background

As a result of continuous improvements in treatment and supportive care, survival rates after childhood cancer have increased over the past decades, now reaching 80% in developed countries. However, the harsh treatment components that have led to increased survival rates can induce serious long-term complications. One in every four childhood cancer survivors (CCS) reveals severe or life-threatening adverse late effects [1], and three in every four survivors report at least one late effect [2, 3]. In female CCS, apart from radiotherapy involving the field of the ovaries or pituitary, alkylating agents are important risk factors for fertility impairment [4–7] and damage is dose-dependent [8]. Such toxic agents can damage the ovarian follicle pool severely, leading to impaired fertility illustrated by an absent or substantially shortened reproductive window. Consequently, considering the current tendency in European countries to postpone childbearing, female survivors may find themselves involuntarily childless, leading to an increased use of artificial reproductive techniques. The feasibility to reach parenthood is of great significance to both parents of children with cancer and to CCS, and is an important determinant of quality of life [9–14]. In addition, gonadal impairment or early menopause carries adverse health risks for women, such as an increased risk for cardiovascular disease and osteoporosis, which require intensive and long-term medical attention [15].

Variations in long-term gonadal impairment in CCS who received the same treatment suggest that genetic variation may be an important determinant of gonadal impairment in CCS. Currently, only limited information is available on the role of genetic factors in the development of impaired gonadal reserve after childhood cancer treatment [4]. One single center study has been performed which evaluated seven genetic single nucleotide polymorphisms (SNPs) in 176 female CCS. These SNPs were selected based on the fact that they have been found to be associated with age at menopause in large genome wide association studies (GWAS) in the general female population [16, 17]. While one of these allelic variations in the *BRSK1* gene (rs1172822) was found associated with a low anti-Müllerian hormone (AMH) level in CCS [4], replication of this finding has not been reported so far. Meanwhile, many more SNPs have been reported to be associated with reproductive ageing in the general population coming from large-scale collaborative consortia [18, 19] but none have yet been investigated in CCS. In order to identify independent genetic determinants for therapy related gonadal impairment, substantially sized cohorts with well-documented clinical as well as treatment data are required. In addition, independent replication cohorts must be available to validate the results. One GWAS [20] has been performed (with Affymetrix 6.0 SNP array) in the St. Jude Lifetime Cohort Study (SJLIFE) among 799 ethnically mixed female CCS, which included an independent replication cohort (genotyped with the Illumina Omni5 SNP array) of 1624 women from the ethnically mixed Childhood Cancer Survivor Study (CCSS). This GWAS did not identify a genome wide significant hit, but found a SNP (rs9999820) that was borderline significantly associated (*p* = 3.3*10^− 7^) with an increased risk of premature menopause, especially in the subgroup of CCS who had undergone ovarian irradiation. This haplotype, consisting of 4 SNPs, is associated with increased hippocampal *NPYR2* gene expresssion, which is associated with a neuroendocrine pathway [20]. Noteworthy is that this GWAS evaluated the genetic variation in (self-reported) premature menopause, the latest manifestation of gonadal impairment or ageing.

The PanCareLIFE initiative, a 5-year (2013–8) EU Framework 7 Programme in the Health Theme originating from the PanCare project, focuses on the identification of determinants of long-term health of CCS. Specifically, PanCareLIFE will evaluate female gonadal impairment, hearing, and quality of life. Investigators from sixteen partner institutions from ten European countries have prospectively and retrospectively collected data from over 12,000 survivors from cancer diagnosed before they were 25 years of age.

The current study is part of this European wide endeavor and focuses on the identification of genetic factors which play a role in the risk of treatment-induced gonadal impairment among female childhood cancer survivors. Its specific objectives are to validate previously identified genetic polymorphisms associated with gonadal impairment in female childhood cancer survivors, using a candidate gene approach; and to identify *novel* SNPs that are independently associated with chemotherapy induced gonadal impairment in female childhood cancer survivors, using a GWAS.

## Methods

### Inclusion criteria

For the current study we included female adult survivors (≥ 18 years) of childhood cancer, diagnosed before the age of 25 years, with a follow-up time of at least 5 years after diagnosis. Eligible survivors had to have been treated with chemotherapy. Exclusion criteria included radiotherapy involving both ovaries, defined as bilateral irradiation of the abdomen below the pelvic crest, or radiotherapy involving the pituitary, defined as cranial or craniospinal irradiation. Furthermore, survivors were not eligible if they had undergone myeloablative allogeneic stem cell transplantation, with or without total body irradiation.

### Study cohort

**Fig. 1:**
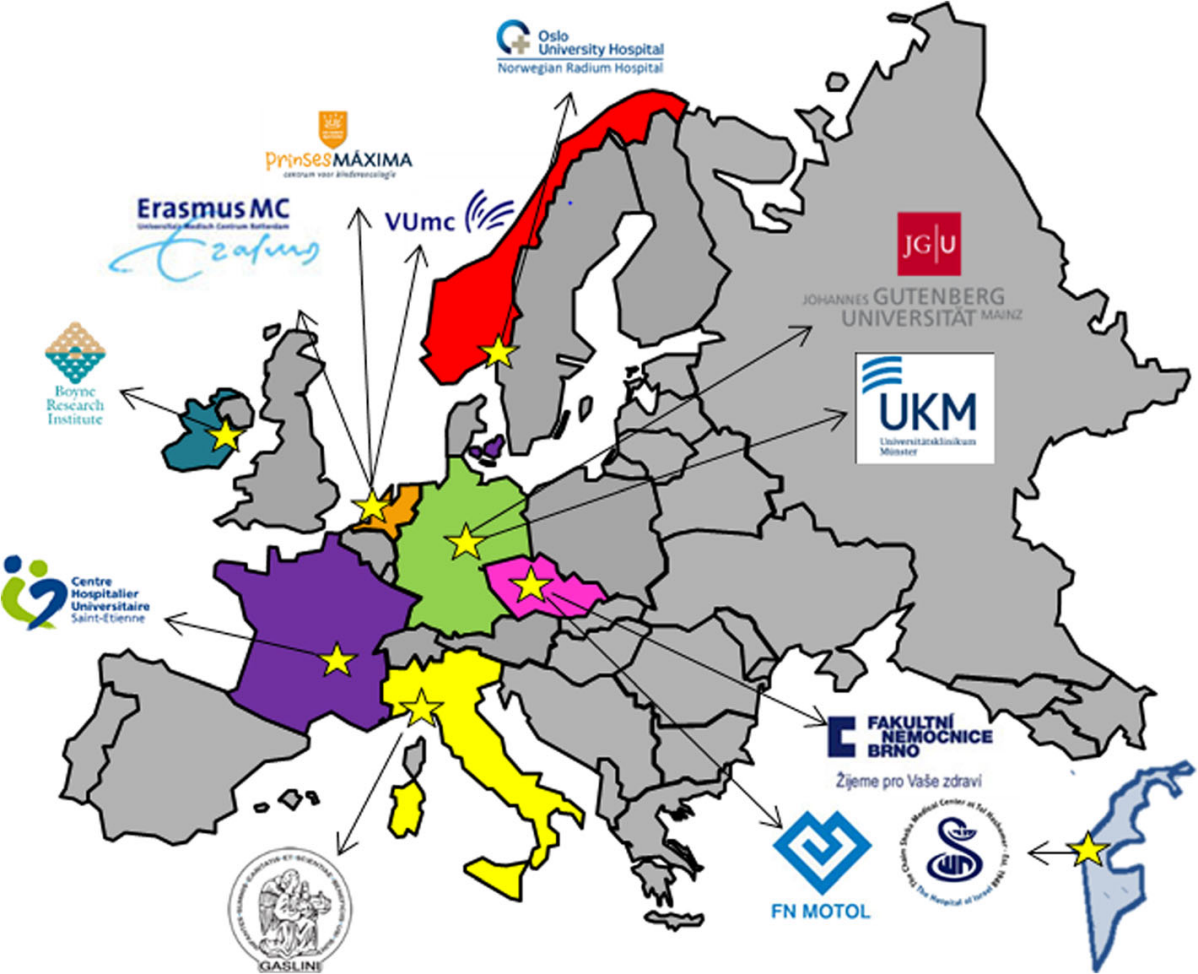
Participating institutions throughout Europe and Israel

PanCareLIFE consists of 8 work packages of which 5 focus on scientific work. Work package 4 encompasses two parts: WP4a focuses on genetic variation in gonadal impairment, and WP4b focuses on genetic variation in ototoxicity. This study addresses work package 4a. For this work package, adult female CCS were recruited in ten institutions from seven countries (Fig. 1). The participating institutions and included numbers were: the Dutch Childhood Oncology Group (AMC, EMC, LUMC, UMCG, UMCN, VUmc) (inclusions *n* = 306), Erasmus Medical Center Rotterdam (*n* = 25) and VU Medical Center Amsterdam (*n* = 19) from the Netherlands, Fakultni Nemocnice Brno (*n* = 134) and Fakultni Nemocnice v Motole (*n* = 86) from Czech Republic, Oslo University Hospital Departments of Oncology/ Pediatrics (*n* = 107) from Norway, I.R.C.C.S. Giannina Gaslini (*n* = 67) from Italy, Department of Paediatric Oncology/University Hospital, St-Etienne (*n* = 64) from France, University Hospital Muenster (*n* = 39) from Germany and Sheba Medical Center (*n* = 18) from Israel. In total 865 CCS were included in this study. DNA samples could not be collected in 28 cases, leaving 837 CCS for analysis (Table 1).

**Table 1:** Data providing institutions in genetic work package on gonadal impairment

Country	Data provider	Treatment data	DNA samples
The Netherlands	Dutch Childhood Oncology Group	306	298
	Erasmus Medical Center, Rotterdam	25	24
	VU Medical Center, Amsterdam	19	18
Czech Republic	Fakultni Nemocnice Brno	134	132
	Fakultni Nemocnice v Motole	86	81
Norway	Oslo University Hospital	107	107
Italy	I.R.C.C.S. Giannina Gaslini	67	64
France	Center Hospitalier Universitaire de Saint-Étienne	64	58
Germany	University Hospital Muenster, Germany	39	37
Israel	Sheba Medical Center, Tel Hashomer	18	18
TOTAL		**865**	**837**

Dutch Childhood Oncology Group: Academic Medical Center in Amsterdam (AMC), Erasmus Medical Center in Rotterdam (EMC), Leiden University Medical Center (LUMC), University Medical Center Groningen (UMCG), University Medical Center Nijmegen (UMCN), VU Medical Center (VUmc)

Medical ethics approval for the study was obtained from all relevant local committees and written informed consent was obtained from all participants.

### Data collection

Basic demographic data of all participants (month and year of birth and of follow-up), diagnostic data (month and year of diagnosis, type of diagnosis) and full details of cancer treatment were retrospectively collected from medical databases and medical records. Data on cancer treatment comprised of details on surgery, chemotherapy and radiotherapy, including start and stop dates and cumulative dosage. All data will be merged at the central data center in Mainz likewise a former EU funded sister project PanCareSurFup [21], and will finally be pseudonymized for the investigators of this study.

### Gonadal function

The primary outcome of this study is AMH level. Serum samples were centrifuged, stored at − 20 °C and shipped on dry ice to the VUmc Amsterdam where all AMH levels were analysed in the same laboratory using an ultra-sensitive Elecsys AMH assay (Roche Diagnostics GmbH, Mannheim, Germany) at one time point. Data on AMH levels were sent to the central data center in Mainz and merged into the central database and subsequently pseudonymized to the investigators. In addition to the continuous AMH levels, patients will be divided in two groups based on AMH levels considered relevant as a proxy for gonadal impairment, considering data on AMH levels in healthy females measured with the same assay in the reference laboratory in the VUmc Amsterdam. These details will be described in detail in the forthcoming manuscript. In addition, detailed information about menstrual history, and/or FSH level, and/or information on usage of artificial reproductive techniques will be used to evaluate gonadal impairment.

### Genotyping

Blood or saliva samples were obtained for DNA isolation. Blood samples (*n* = 781) were stored at ≤ − 20 °C and shipped on dry ice while saliva kits (*n* = 56) were stored and shipped at room temperature. Genomic DNA was extracted by the salting-out method. The choice of genotyping array was made after extensive comparison between all currently available arrays. The Infinium® Global Screening Array was chosen based on the rich up-to-date content and its suitability for GWAS including rare variants, while also containing clinically relevant content, including pharmacogenetics.

### Statistical considerations

For the GWAS a genetic sample size calculation was performed to estimate the number of cases required in the current study [22]. As it is impossible to estimate the allelic frequencies in our population, the following assumptions were made for the power calculation: 1) a high risk allele frequency of 0.2, 2) a genome-wide significant significance level (5*10^− 8^), 3) a cohort size larger than *n* = 800 and 4) a case to control ratio of 1:2. Based on these assumptions, we determined that the number of recruited patients provided statistical power (80%) to identify variants with an odds ratio of at least 1.8.

### Quality control and imputations

A quality control (QC) protocol containing multiple filters will be applied to clean the genetic data and to ensure its quality prior to either imputations or analysis [23]. Both a SNP and individual call rate filter of 97.5% will be applied to remove poorly genotyped SNPs and individuals from the data. Furthermore, a Hardy-Weinberg Equilibrium test (significance level < 1*10^− 7^) will be employed to remove variants containing potential genotyping errors. To ensure sample quality, samples with extreme heterozygosity, gender mismatches, and familial relationships will be assessed and removed. Genetic ancestry of the samples will be assessed and corrected for using principal components (PCs).

Finally, imputations will be performed using the Michigan Imputation Server using default settings [24]. The reference panel chosen for imputations is the Haplotype Reference Consortium (HRC r1.1) [25]. The same approach has previously been used in large-scale population studies such as the Rotterdam Study [26] and Generation R [27].

### Association analysis

For the candidate gene approach we will extract the genotypes of a list of predetermined SNPs based on published literature. The Mann-Whitney U test and the Kruskal-Wallis test will be employed to compare the distribution between groups with continuous data. Logistic regression will be performed to calculate the odds ratio and 95% confidence interval of the SNPs to assess their risk of gonadal impairment. This model will adjust for several confounders: principal component analysis (PCA) will be used to correct for population stratification by modelling ancestry differences between cases and controls [28]. PCA is a common tool that has been widely used for the combined analysis of correlated phenotypes in genetic linkage and association studies [29]. Furthermore, the model will adjust for cyclophosphamide equivalent dose (CED). This measure enables comparison of alkylating agent exposure independent of drug dose distribution within a particular cohort (as the formerly used alkylating agent dose), permitting comparison across different cohorts [30]. In addition, linear regression will be performed to calculate the effect of the SNPs on continuous AMH levels. This model will include age, in addition to the principal components and CED. The modifying effect of genetic predisposition on the association between CED and gonadal impairment will be also explored.

To identify relevant SNPs from the GWAS that may be important but do not reach genome-wide significance, we will use a suggestive significance level of *p* = 5.10^− 6^. After GWAS analysis, we will use the R script EasyQC [31] to clean the association results based, amongst others, on minor allele frequency and imputation quality. The results will then be visualized and the functional annotation for all leading SNPs will be identified using the online platform called Functional Mapping and Annotation of GWAS (FUMA-GWAS) [32].

### Replication

For both the candidate gene approach and GWAS, to ensure that associations are not a chance finding or an artifact due to uncontrolled biases, associations will be replicated within a replication cohort, based on the St. Jude Lifetime Cohort Study (SJLIFE) from St. Jude Children’s Research Hospital, Memphis USA [33, 34] and CCSS cohort.

## Discussion

This paper outlines the design of one study within the PanCareLIFE initiative that has two separate research aims. Female CCS from ten different institutions from seven European countries will be included to validate previously identified genetic polymorphisms associated with gonadal impairment and to identify *novel* SNPs that are independently associated with chemotherapy induced gonadal impairment in female CCS.

Sufficiently-sized cohorts are of key importance in genetic association studies in order to have adequate power to identify low-risk variants. This is especially of importance in the evaluation of common traits such as gonadal function, where many common variants may operate with small effect sizes. To this end, we performed a power calculation to estimate the required cohort size for the current study, based on the estimated allelic frequency in our population.

It is standard practice in current genetic association studies to include an independent replication cohort to validate findings from the initial discovery cohort. However, few large cohorts exist that have sufficient numbers of female CCS, let alone with complete data as well as stored DNA and AMH for analysis. For this project, a collaboration with the St. Jude Children’s Research Hospital, Memphis USA and CCSS has been initiated. AMH levels will be measured in the same laboratory with the same AMH assay for the discovery and replication cohort, thus minimizing lab variation. Given the (non-significant) GWAS observations in the St Jude discovery cohort we believe forces must be joined, and we are therefore actively looking for additional cohorts to include in this and future international collaborations. We encourage readers who are aware of such collections to contact the corresponding author.

Gonadal impairment in CCS can be defined in many ways [35–37], and especially in international collaborations a clear consensus on the definition, as objective as possible, is needed. A separate work package within the PanCareLIFE consortium will combine seven criteria and several different questionnaires to assess clinical gonadal status in 20,000 subjects. For the current study, the primary endpoint AMH was chosen, which will be evaluated both linear as categorized. The secondary endpoint is gonadal impairment based on detailed information about menstrual history, FSH levels and information on usage of artificial reproductive techniques. AMH has the advantage to be as objective as possible, in comparison to questionnaire data that may be prone to recall bias or incorrect information given by the survivor. In addition, AMH can serve as a reliable surrogate marker for ovarian function while the primordial follicle pool is not yet depleted [38, 39]. The only reported GWAS investigating therapy induced fertility impairment in CCS, used premature menopause as primary outcome (clinically assessed in the discovery cohort and self-reported in the replication cohort) [20]. Prior to the clinical manifestation of amenorrhea and increased levels of FSH, impaired gonadal function can be detected by the measurement of lower serum AMH levels [40]. AMH in females is produced solely in the ovary by granulosa cells of small growing follicles and is considered a surrogate marker for ovarian function and ovarian reserve [38, 39]. Like the primordial follicle pool, AMH levels decrease from adolescence on, until menopause occurs. Even survivors who do not report premature menopause (or Primary Ovarian Insufficiency, POI, defined as menopause before the age of 40 years) can still have a poor ovarian function, potentially resulting in reduced fertility or a shorter reproductive window (e.g., early menopause or menopause between 40 and 45 years). This impairment of gonadal function can be identified by the evaluation of AMH levels.

In conclusion, we describe the design of a genetic association study that will evaluate the association of genetic variability with gonadal impairment in a European cohort of childhood cancer survivors, with AMH levels as the primary outcome measure. This international collaboration will enhance knowledge of genetic variation which may be included in risk prediction models for gonadal impairment in CCS. In the future, patients with childhood cancer, parents and survivors may benefit from better individualized counselling concerning future fertility options and necessity for fertility preservation.

## Abbreviations

AMH: Anti-Müllerian hormone
CCS: Childhood cancer survivors
CED: Cyclophosphamide equivalent dose
FSH: Follicle stimulating hormone
GWAS: Genome wide association study
PCA: Principal component analysis
SJLIFE: St. Jude Lifetime Cohort Study
SNP: Single nucleotide polymorphism

## References

[1] Oeffinger KC, Mertens AC, Sklar CA, Kawashima T, Hudson MM, Meadows AT, Friedman DL, Marina N, Hobbie W, Kadan-Lottick NS (2006). Chronic health conditions in adult survivors of childhood cancer. N Engl J Med.

[2] Geenen MM, Cardous-Ubbink MC, Kremer LC, van den Bos C, van der Pal HJ, Heinen RC, Jaspers MW, Koning CC, Oldenburger F, Langeveld NE (2007). Medical assessment of adverse health outcomes in long-term survivors of childhood cancer. Jama.

[3] van Waas M, Neggers SJ, Te Winkel ML, Beishuizen A, Pieters R, van den Heuvel-Eibrink MM: Endocrine late sequelae in long-term survivors of childhood non-Hodgkin lymphoma. Ann Oncol 2012, 23(6):1626–1632. 10.1093/annonc/mdr511 22048153

[4] van Dorp W, van den Heuvel-Eibrink MM, Stolk L, Pieters R, Uitterlinden AG, Visser JA, Laven JS (2013). Genetic variation may modify ovarian reserve in female childhood cancer survivors. Hum Reprod.

[5] Krawczuk-Rybak M, Leszczynska E, Poznanska M, Zelazowska-Rutkowska B, Wysocka J (2013). The progressive reduction in the ovarian reserve in young women after anticancer treatment. Horm Metab Res.

[6] Brougham MF, Crofton PM, Johnson EJ, Evans N, Anderson RA, Wallace WH (2012). Anti-Mullerian hormone is a marker of gonadotoxicity in pre- and postpubertal girls treated for cancer: a prospective study. J Clin Endocrinol Metab.

[7] Lie Fong S, Laven JS, Hakvoort-Cammel FG, Schipper I, Visser JA, Themmen AP, de Jong FH, van den Heuvel-Eibrink MM (2009). Assessment of ovarian reserve in adult childhood cancer survivors using anti-Mullerian hormone. Hum Reprod.

[8] Overbeek A, van den Berg MH, van Leeuwen FE, Kaspers GJL, Lambalk CB, Van Dulmen-den Broeder E (2017). Chemotherapy-related late adverse effects on ovarian function in female survivors of childhood and young adult cancer: a systematic review. Cancer Treat Rev.

[9] Langeveld NE, Grootenhuis MA, Voute PA, de Haan RJ, van den Bos C (2004). Quality of life, self-esteem and worries in young adult survivors of childhood cancer. Psychooncology.

[10] van den Berg H, Repping S, van der Veen F (2007). Parental desire and acceptability of spermatogonial stem cell cryopreservation in boys with cancer. Hum Reprod.

[11] Duffy C, Allen S (2009). Medical and psychosocial aspects of fertility after cancer. Cancer J.

[12] Zebrack BJ, Block R, Hayes-Lattin B, Embry L, Aguilar C, Meeske KA, Li Y, Butler M, Cole S (2013). Psychosocial service use and unmet need among recently diagnosed adolescent and young adult cancer patients. Cancer.

[13] Carter J, Raviv L, Applegarth L, Ford JS, Josephs L, Grill E, Sklar C, Sonoda Y, Baser RE, Barakat RR (2010). A cross-sectional study of the psychosexual impact of cancer-related infertility in women: third-party reproductive assistance. J Cancer Surviv.

[14] Sandrine Thouvenin-Doulet M, Claire Berger, Léonie Casagranda, Odile Oberlin M, Perrine Marec-Berard, Hélène Pacquement, MD, Catherine, Guibout P, Claire Freycon, Tan Dat N’Guyen, Pierre-Yves Bondiau, Delphine Berchery, Chiraz El-Fayech, Béatrice, Trombert-Paviot M, Florent de Vathaire: Fecundity and quality of life of women treated for solid childhood tumours between 1948 and 1992 in France. IN PRINT, JAYAO 2018. 10.1089/jayao.2017.0126 29851372

[15] De Vos M, Devroey P, Fauser BC (2010). Primary ovarian insufficiency. Lancet.

[16] Stolk L, Zhai G, van Meurs JB, Verbiest MM, Visser JA, Estrada K, Rivadeneira F, Williams FM, Cherkas L, Deloukas P (2009). Loci at chromosomes 13, 19 and 20 influence age at natural menopause. Nat Genet.

[17] He C, Kraft P, Chasman DI, Buring JE, Chen C, Hankinson SE, Pare G, Chanock S, Ridker PM, Hunter DJ (2010). A large-scale candidate gene association study of age at menarche and age at natural menopause. Hum Genet.

[18] Day FR, Ruth KS, Thompson DJ, Lunetta KL, Pervjakova N, Chasman DI, Stolk L, Finucane HK, Sulem P, Bulik-Sullivan B (2015). Large-scale genomic analyses link reproductive aging to hypothalamic signaling, breast cancer susceptibility and BRCA1-mediated DNA repair. Nat Genet.

[19] Day FR, Thompson DJ, Helgason H, Chasman DI, Finucane H, Sulem P, Ruth KS, Whalen S, Sarkar AK, Albrecht E (2017). Genomic analyses identify hundreds of variants associated with age at menarche and support a role for puberty timing in cancer risk. Nat Genet.

[20] Brooke RJ, Im C, Wilson CL, Krasin MJ, Liu Q, Li Z, Sapkota Y, Moon W, Morton LM, Wu G, et al. A high-risk haplotype for premature menopause in childhood Cancer survivors exposed to Gonadotoxic therapy. J Natl Cancer Inst. 2018. 10.1093/jnci/djx281 PMC6093389 29432556

[21] Grabow D, Kaiser M, Hjorth L, Byrne J, Alessi D, Allodji RS, Bagnasco F, Bardi E, Bautz A, Bright CJ, et al. The PanCareSurFup cohort of 83,333 five-year survivors of childhood cancer: a cohort from 12 European countries. Eur J Epidemiol. 2018. 10.1007/s10654-018-0370-3 PMC5889790 29497894

[22] Purcell S, Cherny SS, Sham PC (2003). Genetic power calculator: design of linkage and association genetic mapping studies of complex traits. Bioinformatics.

[23] Anderson CA, Pettersson FH, Clarke GM, Cardon LR, Morris AP, Zondervan KT (2010). Data quality control in genetic case-control association studies. Nat Protoc.

[24] Das S, Forer L, Schonherr S, Sidore C, Locke AE, Kwong A, Vrieze SI, Chew EY, Levy S, McGue M (2016). Next-generation genotype imputation service and methods. Nat Genet.

[25] McCarthy S, Das S, Kretzschmar W, Delaneau O, Wood AR, Teumer A, Kang HM, Fuchsberger C, Danecek P, Sharp K (2016). A reference panel of 64,976 haplotypes for genotype imputation. Nat Genet.

[26] Ikram MA, Brusselle GGO, Murad SD, van Duijn CM, Franco OH, Goedegebure A, Klaver CCW, Nijsten TEC, Peeters RP, Stricker BH (2017). The Rotterdam study: 2018 update on objectives, design and main results. Eur J Epidemiol.

[27] Medina-Gomez C, Felix JF, Estrada K, Peters MJ, Herrera L, Kruithof CJ, Duijts L, Hofman A, van Duijn CM, Uitterlinden AG (2015). Challenges in conducting genome-wide association studies in highly admixed multi-ethnic populations: the generation R study. Eur J Epidemiol.

[28] Chan YH (2004). Biostatistics 302. Principal component and factor analysis. Singap Med J.

[29] Ma S, Dai Y (2011). Principal component analysis based methods in bioinformatics studies. Brief Bioinform.

[30] Green DM, Nolan VG, Goodman PJ, Whitton JA, Srivastava D, Leisenring WM, Neglia JP, Sklar CA, Kaste SC, Hudson MM (2014). The cyclophosphamide equivalent dose as an approach for quantifying alkylating agent exposure: a report from the childhood Cancer survivor study. Pediatr Blood Cancer.

[31] Winkler TW, Day FR, Croteau-Chonka DC, Wood AR, Locke AE, Magi R, Ferreira T, Fall T, Graff M, Justice AE (2014). Quality control and conduct of genome-wide association meta-analyses. Nat Protoc.

[32] Watanabe K, Taskesen E, van Bochoven A, Posthuma D: Functional mapping and annotation of genetic associations with FUMA. Nat Commun 2017, 8(1):1826. 10.1038/s41467-017-01261-5 PMC5705698 29184056

[33] Hudson MM, Ness KK, Nolan VG, Armstrong GT, Green DM, Morris EB, Spunt SL, Metzger ML, Krull KR, Klosky JL (2011). Prospective medical assessment of adults surviving childhood cancer: study design, cohort characteristics, and feasibility of the St. Jude lifetime cohort study. Pediatr Blood Cancer.

[34] Hudson MM, Ehrhardt MJ, Bhakta N, Baassiri M, Eissa H, Chemaitilly W, Green DM, Mulrooney DA, Armstrong GT, Brinkman TM (2017). Approach for classification and severity grading of long-term and late-onset health events among childhood Cancer survivors in the St. Jude lifetime cohort. Cancer Epidemiol Biomark Prev.

[35] Barton SE, Najita JS, Ginsburg ES, Leisenring WM, Stovall M, Weathers RE, Sklar CA, Robison LL, Diller L (2013). Infertility, infertility treatment, and achievement of pregnancy in female survivors of childhood cancer: a report from the childhood Cancer survivor study cohort. Lancet Oncol.

[36] Green DM, Kawashima T, Stovall M, Leisenring W, Sklar CA, Mertens AC, Donaldson SS, Byrne J, Robison LL (2009). Fertility of female survivors of childhood cancer: a report from the childhood cancer survivor study. J Clin Oncol.

[37] van der Kooi AL, Van den Heuvel-Eibrink MM, van Noortwijk A, Neggers SJ, Pluijm SM, van Dulmen-den Broeder E, van Dorp W, Laven JS (2017). Longitudinal follow-up in female childhood Cancer survivors: no signs of accelerated ovarian function loss. Hum Reprod.

[38] de Vet A, Laven JSE, de Jong FH, Themmen APN, Fauser BCJM (2002). Antimüllerian hormone serum levels: a putative marker for ovarian aging. Fertil Steril.

[39] Rosen MP, Johnstone E, McCulloch CE, Schuh-Huerta SM, Sternfeld B, Reijo-Pera RA, Cedars MI (2012). A characterization of the relationship of ovarian reserve markers with age. Fertil Steril.

[40] van Beek RD, van den Heuvel-Eibrink MM, Laven JSE, de Jong FH, Themmen APN, Hakvoort-Cammel FG, van den Bos C, van den Berg H, Pieters R, de Muinck Keizer-Schrama SM. Anti-Müllerian hormone is a sensitive serum marker for gonadal function in women treated for Hodgkin’s lymphoma during childhood. J Clini Endocrinol Metabol. 2007;92(10):3869–74. 10.1210/jc.2006-2374 17726078

